# Bis(4-nitro­phen­yl) selenide

**DOI:** 10.1107/S1600536813007526

**Published:** 2013-04-05

**Authors:** Zong-Le Zuo

**Affiliations:** a Chemical Synthesis and Pollution Control Key Laboratory of Sichuan Province, China West Normal University, Nanchong 637002, People’s Republic of China

## Abstract

In the title compound, C_12_H_8_N_2_O_4_Se, the Se atom is situated on a twofold rotational axis, so the asymmetric unit contains one half-mol­ecule. In the mol­ecule, the C—Se—C angle is 99.48 (13)°, the two benzene rings are inclined to each other at an angle of 63.8 (1)° and the nitro group is twisted by 15.9 (1)° from the attached benzene ring. In the crystal, mol­ecules are held together through weak C—H⋯O inter­actions, forming a three-dimensional network.

## Related literature

For applications of organoselenium compounds, see: Mugesh *et al.* (2001 ▶); Nogueira *et al.* (2004 ▶); Wirth (1999 ▶). For details of the synthesis, see: Taniguchi (2005 ▶). The crystal structures of the related compounds bis­(p-tol­yl) selenide and bis­(4-acetyl­phen­yl) selenide were reported by Blackmore & Abrahams (1955 ▶) and Bouraoui *et al.* (2011 ▶), respectively.
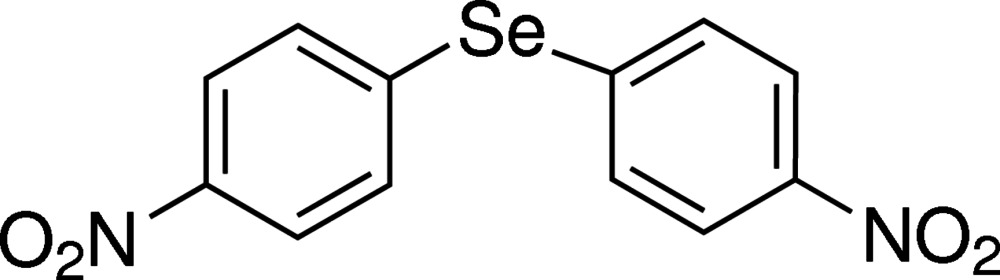


## Experimental

### 

#### Crystal data

C_12_H_8_N_2_O_4_Se
*M* *_r_* = 323.16
Monoclinic, 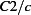

*a* = 7.207 (4) Å
*b* = 14.176 (7) Å
*c* = 11.686 (5) Å
β = 101.870 (7)°
*V* = 1168.3 (9) Å^3^
*Z* = 4
Mo *K*α radiation
μ = 3.23 mm^−1^
*T* = 153 K
0.47 × 0.34 × 0.34 mm

#### Data collection

Rigaku AFC10/Saturn724+ diffractometer
Absorption correction: multi-scan (*CrystalClear*; Rigaku, 2008 ▶) *T*_min_ = 0.314, *T*_max_ = 0.402
4848 measured reflections
1557 independent reflections
1298 reflections with *I* > 2σ(*I*)
*R*_int_ = 0.036

#### Refinement

*R*[*F* ^2^ > 2σ(*F* ^2^)] = 0.030
*wR*(*F* ^2^) = 0.074
*S* = 1.00
1557 reflections
87 parameters
H-atom parameters constrained
Δρ_max_ = 0.70 e Å^−3^
Δρ_min_ = −0.50 e Å^−3^

### 

Data collection: *CrystalClear* (Rigaku, 2008 ▶); cell refinement: *CrystalClear*; data reduction: *CrystalClear*; program(s) used to solve structure: *SHELXS97* (Sheldrick, 2008 ▶); program(s) used to refine structure: *SHELXL97* (Sheldrick, 2008 ▶); molecular graphics: *SHELXTL* (Sheldrick, 2008 ▶); software used to prepare material for publication: *SHELXTL*.

## Supplementary Material

Crystal structure: contains datablock(s) global, I. DOI: 10.1107/S1600536813007526/cv5389sup1.cif

Structure factors: contains datablock(s) I. DOI: 10.1107/S1600536813007526/cv5389Isup2.hkl

Supplementary material file. DOI: 10.1107/S1600536813007526/cv5389Isup3.cml

Additional supplementary materials:  crystallographic information; 3D view; checkCIF report

## Figures and Tables

**Table 1:** Hydrogen-bond geometry (Å, °)

*D*—H⋯*A*	*D*—H	H⋯*A*	*D*⋯*A*	*D*—H⋯*A*
C2—H2⋯O2^i^	0.95	2.51	3.426 (3)	162
C6—H6⋯O2^ii^	0.95	2.50	3.427 (3)	164

Symmetry codes: (i) 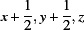; (ii) 
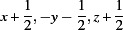
.

**Fig. 1.**
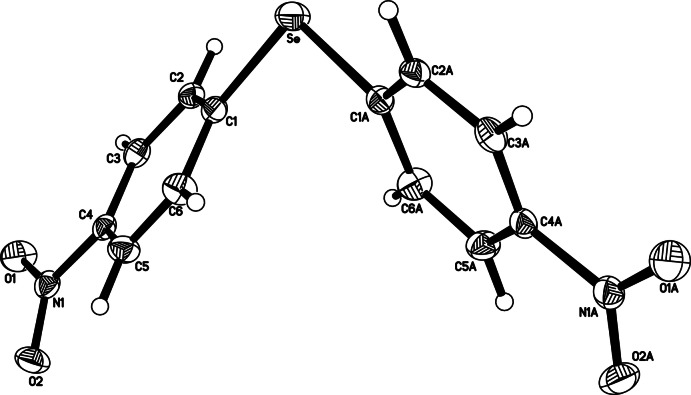
The molecular structure of (I) showing 50% probability displacement ellipsoids and the atomic numbering [symmetry code: (A) -x, y, 3/2-z].

## supplementary crystallographic information

### Comment

During the past decade, organoselenium compounds have emerged their potential as drug candidates (Nogueira *et al.*, 2004; Mugesh *et al.*, 2001). Organoselenium compounds also exert catalytic role in organic synthesis (Wirth, 1999). Herewith we report the crystal structure of the title compound, (I).

In (I) (Fig. 1), each Se atom is situated on a twofold rotational axis, so asymmetric part contains a half of the molecule. The C—Se—C angle is 99.48 (13)°, that is similar to 99.58 (13)° observed in bis(4-acetylphenyl) selenide (Bouraoui *et al.*, 2011), but different from 106.2 (1)° found in bis(*p*-tolyl) selenide (Blackmore & Abrahams, 1955). Two benzene rings in (I) are inclined to each other at 63.8 (1)° and each nitro group is twisted at 15.9 (1)° from the attached benzene ring. The crystal packing is stabilized by the weak C—H···O hydrogen bonds (Table 1).

### Experimental

The title compound has been synthesized following the procedure proposed by Taniguchi (2005). 1-Iodo-4-nitrobenzene (1.0 mmol), selenium (1.2 mmol), cuprous oxide (0.1 mmol), aluminium (2 mmol), magnesium chloride (0.5 mmol), acetylacetone (0.3 mmol), TBAF (0.2 mmol) and DMF/water (3:1, 1.0 ml) were put into a Teflon septum screw-capped tube and then sealed in the air. The reaction mixture was stirred at 120 °C for 36 h, then cooled to room temperature. Subsequently, the resulting mixture was diluted with ethyl acetate and water, and the combined organic extracts were dried with sodium sulfate anhydrous. After the solvent being removed under reduced pressure, the residue was purified by silica-gel column chromatography to afford the corresponding product. Yellow single crystals suitable for X-ray diffraction were obtained by recrystallization from acetone.

### Refinement

H atoms were placed in calculated positions, with C—H = 0.95 Å, and refined using a riding model, with *U*_iso_(H) = 1.2*U*_eq_(C).

### Crystal data

**Table:**

C_12_H_8_N_2_O_4_Se	*F*(000) = 640
*M**_r_* = 323.16	*D*_x_ = 1.837 Mg m^−^^3^
Monoclinic, *C*2/*c*	Mo *K*α radiation, λ = 0.71073 Å
Hall symbol: -C 2yc	Cell parameters from 1957 reflections
*a* = 7.207 (4) Å	θ = 2.9–29.1°
*b* = 14.176 (7) Å	µ = 3.23 mm^−^^1^
*c* = 11.686 (5) Å	*T* = 153 K
β = 101.870 (7)°	Block, yellow
*V* = 1168.3 (9) Å^3^	0.47 × 0.34 × 0.34 mm
*Z* = 4	

### Data collection

**Table:**

Rigaku AFC10/Saturn724+ diffractometer	1557 independent reflections
Radiation source: fine-focus sealed tube	1298 reflections with *I* > 2σ(*I*)
Graphite monochromator	*R*_int_ = 0.036
Detector resolution: 28.5714 pixels mm^-1^	θ_max_ = 29.1°, θ_min_ = 3.2°
phi and ω scans	*h* = −9→9
Absorption correction: multi-scan (*CrystalClear*; Rigaku, 2008)	*k* = −19→16
*T*_min_ = 0.314, *T*_max_ = 0.402	*l* = −15→11
4848 measured reflections	

### Refinement

**Table:**

Refinement on *F*^2^	Primary atom site location: structure-invariant direct methods
Least-squares matrix: full	Secondary atom site location: difference Fourier map
*R*[*F*^2^ > 2σ(*F*^2^)] = 0.030	Hydrogen site location: inferred from neighbouring sites
*wR*(*F*^2^) = 0.074	H-atom parameters constrained
*S* = 1.00	*w* = 1/[σ^2^(*F*_o_^2^) + (0.0351*P*)^2^ + 0.160*P*] where *P* = (*F*_o_^2^ + 2*F*_c_^2^)/3
1557 reflections	(Δ/σ)_max_ < 0.001
87 parameters	Δρ_max_ = 0.70 e Å^−^^3^
0 restraints	Δρ_min_ = −0.50 e Å^−^^3^

### Special details

**Table:**

Geometry. All e.s.d.'s (except the e.s.d. in the dihedral angle between two l.s. planes) are estimated using the full covariance matrix. The cell e.s.d.'s are taken into account individually in the estimation of e.s.d.'s in distances, angles and torsion angles; correlations between e.s.d.'s in cell parameters are only used when they are defined by crystal symmetry. An approximate (isotropic) treatment of cell e.s.d.'s is used for estimating e.s.d.'s involving l.s. planes.
Refinement. Refinement of *F*^2^ against ALL reflections. The weighted *R*-factor *wR* and goodness of fit *S* are based on *F*^2^, conventional *R*-factors *R* are based on *F*, with *F* set to zero for negative *F*^2^. The threshold expression of *F*^2^ > σ(*F*^2^) is used only for calculating *R*-factors(gt) *etc*. and is not relevant to the choice of reflections for refinement. *R*-factors based on *F*^2^ are statistically about twice as large as those based on *F*, and *R*- factors based on ALL data will be even larger.

### Fractional atomic coordinates and isotropic or equivalent isotropic displacement parameters (Å^2^)

**Table:**

	*x*	*y*	*z*	*U*_iso_*/*U*_eq_	
Se	0.0000	0.05282 (2)	0.7500	0.02293 (12)	
O1	−0.6302 (3)	−0.18522 (11)	0.34112 (15)	0.0281 (4)	
O2	−0.5748 (2)	−0.29360 (11)	0.47417 (15)	0.0251 (4)	
N1	−0.5486 (3)	−0.21464 (13)	0.43644 (16)	0.0182 (4)	
C1	−0.1638 (3)	−0.03449 (14)	0.65264 (19)	0.0162 (4)	
C2	−0.2197 (3)	−0.01493 (15)	0.53474 (19)	0.0166 (4)	
H2	−0.1711	0.0392	0.5030	0.020*	
C3	−0.3464 (3)	−0.07375 (15)	0.4622 (2)	0.0176 (5)	
H3	−0.3873	−0.0601	0.3813	0.021*	
C4	−0.4114 (3)	−0.15292 (14)	0.51142 (19)	0.0157 (4)	
C5	−0.3547 (3)	−0.17464 (16)	0.6281 (2)	0.0195 (5)	
H5	−0.3994	−0.2303	0.6588	0.023*	
C6	−0.2320 (3)	−0.11472 (15)	0.70005 (19)	0.0198 (5)	
H6	−0.1942	−0.1279	0.7812	0.024*	

### Atomic displacement parameters (Å^2^)

**Table:**

	*U*^11^	*U*^22^	*U*^33^	*U*^12^	*U*^13^	*U*^23^
Se	0.02479 (19)	0.01636 (18)	0.0236 (2)	0.000	−0.00441 (13)	0.000
O1	0.0308 (10)	0.0249 (9)	0.0228 (9)	0.0009 (7)	−0.0082 (7)	−0.0017 (7)
O2	0.0284 (10)	0.0164 (8)	0.0296 (10)	−0.0054 (7)	0.0037 (8)	−0.0012 (7)
N1	0.0158 (10)	0.0187 (10)	0.0197 (10)	0.0021 (7)	0.0024 (8)	−0.0048 (7)
C1	0.0145 (10)	0.0153 (11)	0.0180 (11)	0.0013 (8)	0.0016 (8)	−0.0020 (8)
C2	0.0163 (10)	0.0147 (10)	0.0191 (12)	0.0014 (8)	0.0039 (9)	0.0038 (8)
C3	0.0174 (11)	0.0205 (12)	0.0142 (11)	0.0038 (8)	0.0016 (8)	0.0019 (8)
C4	0.0130 (10)	0.0154 (11)	0.0182 (11)	0.0023 (8)	0.0016 (8)	−0.0045 (8)
C5	0.0223 (12)	0.0160 (11)	0.0199 (12)	−0.0010 (9)	0.0038 (9)	0.0031 (8)
C6	0.0245 (12)	0.0205 (12)	0.0134 (11)	−0.0014 (9)	0.0018 (9)	0.0026 (8)

### Geometric parameters (Å, º)

**Table:**

Se—C1	1.915 (2)	C2—H2	0.9500
Se—C1^i^	1.915 (2)	C3—C4	1.386 (3)
O1—N1	1.221 (2)	C3—H3	0.9500
O2—N1	1.232 (2)	C4—C5	1.375 (3)
N1—C4	1.468 (3)	C5—C6	1.380 (3)
C1—C2	1.382 (3)	C5—H5	0.9500
C1—C6	1.398 (3)	C6—H6	0.9500
C2—C3	1.389 (3)		
			
C1—Se—C1^i^	99.48 (13)	C4—C3—H3	121.0
O1—N1—O2	123.79 (19)	C2—C3—H3	121.0
O1—N1—C4	118.68 (18)	C5—C4—C3	122.4 (2)
O2—N1—C4	117.53 (18)	C5—C4—N1	118.99 (19)
C2—C1—C6	120.3 (2)	C3—C4—N1	118.59 (19)
C2—C1—Se	118.71 (16)	C4—C5—C6	119.3 (2)
C6—C1—Se	120.96 (17)	C4—C5—H5	120.4
C1—C2—C3	120.6 (2)	C6—C5—H5	120.4
C1—C2—H2	119.7	C5—C6—C1	119.5 (2)
C3—C2—H2	119.7	C5—C6—H6	120.2
C4—C3—C2	117.9 (2)	C1—C6—H6	120.2
			
C1^i^—Se—C1—C2	−139.8 (2)	O2—N1—C4—C5	−15.9 (3)
C1^i^—Se—C1—C6	42.66 (16)	O1—N1—C4—C3	−14.9 (3)
C6—C1—C2—C3	1.0 (3)	O2—N1—C4—C3	165.32 (19)
Se—C1—C2—C3	−176.55 (16)	C3—C4—C5—C6	1.5 (3)
C1—C2—C3—C4	−1.2 (3)	N1—C4—C5—C6	−177.24 (19)
C2—C3—C4—C5	−0.1 (3)	C4—C5—C6—C1	−1.7 (3)
C2—C3—C4—N1	178.67 (18)	C2—C1—C6—C5	0.4 (3)
O1—N1—C4—C5	163.9 (2)	Se—C1—C6—C5	177.94 (17)

Symmetry code: (i) −*x*, *y*, −*z*+3/2.

### Hydrogen-bond geometry (Å, º)

**Table:**

*D*—H···*A*	*D*—H	H···*A*	*D*···*A*	*D*—H···*A*
C2—H2···O2^ii^	0.95	2.51	3.426 (3)	162
C6—H6···O2^iii^	0.95	2.50	3.427 (3)	164

Symmetry codes: (ii) *x*+1/2, *y*+1/2, *z*; (iii) *x*+1/2, −*y*−1/2, *z*+1/2.
